# A Heuristic Placement Selection of Live Virtual Machine Migration for Energy-Saving in Cloud Computing Environment

**DOI:** 10.1371/journal.pone.0108275

**Published:** 2014-09-24

**Authors:** Jia Zhao, Liang Hu, Yan Ding, Gaochao Xu, Ming Hu

**Affiliations:** 1 College of Computer Science and Engineering, ChangChun University of Technology, Changchun, China; 2 College of Computer Science and Technology, Jilin University, Changchun, China; Shenzhen Institutes of Advanced Technology, China

## Abstract

The field of live VM (virtual machine) migration has been a hotspot problem in green cloud computing. Live VM migration problem is divided into two research aspects: live VM migration mechanism and live VM migration policy. In the meanwhile, with the development of energy-aware computing, we have focused on the VM placement selection of live migration, namely live VM migration policy for energy saving. In this paper, a novel heuristic approach PS-ES is presented. Its main idea includes two parts. One is that it combines the PSO (particle swarm optimization) idea with the SA (simulated annealing) idea to achieve an improved PSO-based approach with the better global search's ability. The other one is that it uses the Probability Theory and Mathematical Statistics and once again utilizes the SA idea to deal with the data obtained from the improved PSO-based process to get the final solution. And thus the whole approach achieves a long-term optimization for energy saving as it has considered not only the optimization of the current problem scenario but also that of the future problem. The experimental results demonstrate that PS-ES evidently reduces the total incremental energy consumption and better protects the performance of VM running and migrating compared with randomly migrating and optimally migrating. As a result, the proposed PS-ES approach has capabilities to make the result of live VM migration events more high-effective and valuable.

## Introduction

In green cloud computing, live VM migration technology [1] has always been playing a critical role. It not only contributes to implement cloud computing systems such as Infrastructure as a Service architecture [2], [3] but also is the embody of elasticity and flexibility of the green cloud computing idea. Also, the maintenance management can be achieved through live VM migration technology in green cloud computing data centers, in which there exist many occasions needing live VM migrtion events. For instance, all VMs of some host probably need to be migrated out on account of a shut-down requirement of the host. Some VMs of a host probably need to be migrated out because of a load balancing requirement, a re-allocation requirement or other goals etc. Whenever a VM is ready to be migrated, generally there are large number of available hosts which can accommodate it and meet its resource requirements in the current cloud data centers. Traditionally, the migration target of the VM will be chosen randomly from these available hosts and then one can automatically or manually move the VM to the target host. It is obviously that the way to randomly selecting a target host for a live VM migration isn't efficient in any way.

In the contemporary society, large number of data centers working worldwide are having huge energy consumption, whose impact on environments is evident and considerable. Many researchers have been attempting to propose effective approaches with energy consumption of data centers minimized while ensuring the desired QoS (Quality of Service). With the rapid development of cloud computing and green computing, an array of researchers have focused their attention on green cloud data centers based on cloud computing and virtualized technologies and aiming to minimize energy consumption. This paper has focused on live VM migration policy for energy saving in this context of green cloud data centers. In virtualized green cloud data centers, there are always plenty of VMs needing to be migrated for certain goals. These migrant VMs, however, have many valid target hosts to select from. It is generally acknowledged that only one target host is most suitable for the VM in the aspect of minimizing the total incremental energy consumption in cloud data center. It is the proposed objective to pick out the optimal target hosts of migrant VMs.

Varieties of existing works have presented some heuristic algorithms to find optimal solutions aiming to minimize energy consumption. The basic idea is that the controllers have searched for a best policy by using their proposed algorithms on the basis of the current situation and history of a cloud data center. The problems of convergence and local optimization have been challenging the research direction. On the other hand, we know that a data center doesn't have abilities in predicting the size and type of the next workloads. As a consequence, the optimal policy which the proposed algorithms have found out over a short period of time isn't necessarily the optimal solution over a long period of time. In a word, the global best which of some VM the proposed algorithms have found out in an algorithm cycle may be a local best in a long-term process. Moreover, since the capability with which the current random migration policy and optimal migration policy adapt to a dynamic cloud environment isn't excellent enough, they may cause many failure events of live VM migration in a real and dynamic cloud environment. To address these problems, this paper has put forward a novel heuristic approach PS-ES, which employs the improved PSO method and utilizes the idea of SA to achieve a long-term energy saving optimization. Besides, a parameter, which can make the proposed PS-ES have capability to self-adapt to a dynamic cloud environment, is introduced into PS-ES to improve the performance of live VM migration. That is, the failure rate of VM migration is decreased. Compared to migrating a VM randomly and optimally, the proposed PS-ES has decreased more incremental energy consumption and failure events of live VM migration to contribute to achieving better green cloud data centers.

The rest of the paper is organized as follows. In Section 2, we present the related work of our proposed approach aiming to green cloud data center and the reasonable prerequisites are shown clearly. In Section 3, the analysis of the problem proposed in this paper and its formulation are given. In Section 4, the algorithm and implementation of PS-ES are introduced in detail. In Section 5, the experimental results and analysis on CloudSim platform are given. Finally, in Section 6, we summarize the full paper and future work is put forward.

## Related Work

At present, the proposed problem concerning finding an appropriate target host for a live VM migration in terms of the objective of minimizing the total incremental energy consumption from the perspective of a long period of time has not been widely researched in the related fields. Most researchers, however, have focused on some problems similar to the proposed problem in this paper. Some researchers have focused on the direction targeting other problems of minimizing the incremental energy consumption by utilizing the technology of live VM migration in cloud data centers. Similarly, some researchers have been studying the direction which utilizes live VM migration technology to move these VMs in order to fulfill the requirement of performance and workload limitation while minimizing the energy drawn by a cloud data center. Most of them, in fact, are just to find an optimal host for each VM, which will be migrated or be created in it with the energy drawn by cloud data centers minimized. Ergo, the related work of the kind of problems of green cloud data centers will be discussed briefly in this section.

Rusu et al. in [4] have proposed a novel energy saving and cluster-level appraoch, which has abilities in dynamically reconfiguring the cluster to reduce energy consumption in the case that the load is decreased. The proposed appraoch includes two important components: the front end manager and the local manager. The front end manager is in charge of monitoring the cluster consisting of many hosts. In terms of a given system load, when the front end manager finds that some hosts should be turned on or off, the local managers of the corresponding hosts will take advantage of DVFS (Dynamic Voltage and Frequency Scaling) technology to save energy. The approach depends on the table of values and needs computing offline. Besides, the proposed system should have made use of consolidation technology through live VM migration and thus to duly turn off some hosts for better energy saving. As a result, its on/off policy and performance may not be effective enough.

In literature [5], Srikantaiah et al. have focused on the problem of dynamic consolidation of applications used for serving small stateless requests in cloud data centers to minimize energy consumption. The proposed problem is abstracted as a multi-dimensional bin packing problem. The authors have proposed a heuristic approach to address the defined bin packing problem. Its main idea is to minimize the sum of the Euclidean distances between the current allocations to the optimal point at each host. By using the proposed approach, the application workload will be allocated to a host once a request to executing a new application is received. If all active hosts do not have capabilities enough to process the current workload, a new host will be switch on while re-allocating all the workloads by utilizing the proposed approach in an arbitrary order. The proposed approach is suitable for heterogeneous environments. However, there exist some deficiencies in the proposed approach. It can only work well under the circumstances that the resource requirements of all applications are known in advance and can't be changed. The performance and energy overhead not considered by the authors are caused by migration of state-full applications between nodes. Besides, the frequent switching servers on/off also results in significant costs which are not negligible for a real-world system.

In literature [6], Verma et al. has focused on reducing energy consmpution of cloud data centers by utilizing application placement achieved by live VM migration while ensuring minimizing migration cost. A novel application placement architecture pMapper is proposed by the authors. In pMapper, there are three major components given. A performance manager is used to dynamically resize VMs. A energy manager is achieved through CPU throttling. And a migration manager is in charge of identifying the target host for migration by using a knowledge base. The authors have presented that it is not necessary for energy-aware scheduling approaches to estimate energy consumption values. As long as the scheduling algorithms have capabilities to pick out the host which can relatively minimize the total incremental energy consumption due to a new VM being placed, it can schedule the given VM to the fit host. The proposed pMapper architecture minimizes energy and migration costs with keeping the performance. Our approach is based on a heuristic approach which exploits the concept of minimizing the total incremental energy owing to the new VM migrations. Our proposed architecture is simple and doesn't need any knowledge base to achieve significant reduction in the energy consumption.

Bo Li et al. in [7] have presented a novel approach EnaCloud. It has aimed to achieve a energy-efficient cloud platform by utilizing live application placement. Like pMapper, the application is also encapsulated in a VM in EnaCloud to support its live migration to make the number of running hosts minimized in order to save energy. EnaCloud has modeled the VM placement problem as a bin packing problem and proposed a heuristic algorithm to obtain the better solution. Besides, a novel over-provision method is presented to deal with the varying resource requirements of VMs encapsulating applications. Although this method can reduce extra operation overhead due to the changes of resource demands, it has risk to optimizing this problem. That is, it has possibility to result in more overhead and cost.

Jeyarani et al. [8] have proposed SAPSO (self-adaptive particle swarm optimization) for efficient virtual machine provisioning in cloud aimed at that when mapping a set of VM instances onto a set of servers from a dynamic resource pool, the total incremental energy drawn upon the mapping is minimal and does not compromise the performance objectives. The advantage of the proposed solution is obvious. It has focused on not only improving the performance of workload facilitating the cloud consumers but also developing the energy efficient data center management to facilitate cloud providers. However, the approach still may be inefficient and cause some additional events and costs from a long-term perspective as it doesn't take the future workload into account. Our proposed algorithm PS-ES is a heuristic approach which is based on PSO, one of swarm intelligence algorithms and introduces the SA (Simulated Annealing) idea into it.

Gaochao Xu et al. [9] present a novel heuristic approach named PS-ABC. Its algorithm consists of two parts. One part is that it combines the artificial bee colony (ABC) idea with the uniform random initialization idea, the binary search idea, and Boltzmann selection policy to achieve an improved ABC-based approach with better global exploration's ability and local exploitation's ability. The other part is that it uses the Bayes theorem to further optimize the improved ABC-based process to faster get the final optimal solution. The whole approach achieves a longer-term efficient optimization for power saving. However, it is by achieving the more accurate solution of the current problem window that its longer-term energy saving optimization effect is reached. It doesn't take the whole problem into consideration from the long-term operation perspective of a cloud data center. Although it has relatively achieved the global optimization in an algorithm cycle, it may get a local optimal result from the long-term operation of cloud data centers. As a result, the proposed PS-ES in this paper has the better energy saving effect than PS-ABC from a long-term operation of cloud data centers.

In this paper, PS-ES has a prerequisite. PS-ES aims to pick out the target host of each of the n migrant VMs from all m available hosts. It is assumed that each VM's target host picked out by PS-ES will be not the host that the VM is moved out from. The algorithm provides a live VM migration policy aiming to green cloud data centers. Therefore, the fact that the VM should be moved out for its reason is the premise of our approach. Since a VM needs to be migrated from its source host, its candidate hosts will not include its source host. Otherwise, it does not need the current migration event. What is more is that a host both needs move out VMs while having ability in receiving VMs is impossible and non-objective within a time window Δt. It can be seen that, for all the migrant VMs, the hosts, each of which is the source host of some migrant VM, will not be the target hosts. Therefore, the proposed prerequisite is rational and natural. The performance and efficiency of PS-ES will not be affected by it.

## Results and Discussion

### 1. The Proposed Problem

In cloud data centers based on the IaaS architecture, the running VMs always have some requirements of live migration for some reasons in physical hosts. Determing the target host is the indispensable step of live VM migration. And the number of the available target hosts of a migrant VM is more than one. Besides, the varying placement selections of available target hosts will also lead to the different energy consumption. As a consequence, it is necessary for IaaS cloud data centers based on virtualized technologies to have a high-efficient energy saving placement selection policy for migrating the migrant VMs into the appropriate physical hosts. In this paper, the proposed problem is the live migration policy problem of VM placement selection of available target physical hosts.

### 2. Problem Formulation

The proposed problem can be formulated as migrating n VMs accumulated within a time window **Δt** into m available candidate physical hosts. An n-dimensional vector is utilized to represent a solution of the proposed problem. The target host No. of the migrant VM which the location of an element of the vector represents is the value on the element. It is assumed that there exist m available physical hosts in the resource pool of the cloud data center for the current problem window. The m hosts are heterogeneous and dynamic while using space shared allocation policy. The states of the hosts are changed dynamically in real time in the light of the corresponding workloads. The problem can be stated as follows. Search out a VM-host set **PS** of placement selections so as to minimize the total incremental energy consumption caused by the migrated VMs onto the corresponding physical hosts while maximizing the performance by fulfilling the resource requirements of maximum number of VMs. A four tuple ***S*** **= {PH, VM, E, PS}** for the proposed problem scenario is defined. **PH** is a set of **m** available physical hosts denoted by **PH (m, t) = {PH_1_**, **PH_2_**, **PH_3_ …**, **PH_m_}**, available at migrating start time **t**. **VM** is a set of **n** VMs denoted by **VM (n, t, Δt) = {VM_1_**,**VM_2_**, **VM_3_**, **…**, **VM_n_}** accumulated within a time window **Δt**. **E (m, t) = {E_1_**, **E_2_**, **…**, **E_m_}** is the energy consumption by the **m** physical hosts in resource pool. It is obvious that the proposed problem has a host of solutions which meet the performance constraints of the VM requests. That is, it is a multimodal problem having more than one placement selection to select from. Consequently, finding all of **o** candidate placement selections capable of maximizing the performance and then finding which among minimizes the energy consumption are supposed to be the objective. In the first place, a metric *τ* ***_r_*** denoting resource fulfillment requirement is defined as follows to meet the performance demand above all.
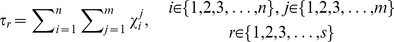
(1)where 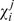 marks the placement selection of ***i***th VM on the ***j***th host and is defined as following. The total number of placement selections is represented as **s**.
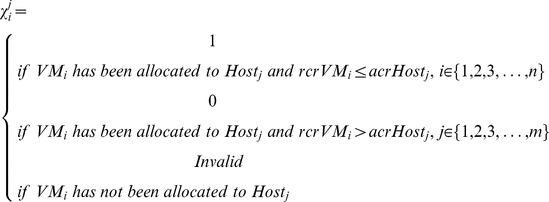
(2)where the minimized computing resource requirements of ***i***th VM and the available computing resource of ***j***th host are represented by **rcrVM_i_** and **acrHost_j_** respectively. **o** placement selections with maximum *τ* ***_r_*** values are picked out from all **s** placement selections and **PS [m, n, o, t]** denotes them.

The next metric is designed on the energy consumption. Let **PS [m, n, r, t+t_0_** **(** ***k*** **)]** represent the migration of successive VMs, **r** represents any one in **o** placement selections and an integer **k** is utilized to represent a stage, increasing with successive migrating. A reasonable case in point may be that if **k** is 5, the fifth VM can be migrated to the host marked at the fifth location of the **r** vector through the proposed appraoch. **PS [m, n, r, t+t_0_** **(k)]** **r** 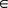 **{1,2,3,…,o}** denotes the **o** placement selections at stage **k** and 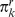 representes the corresponding energy consumption. It means that, having migrated the ***k***th VM to its target host in terms of **r** placement vector, the total energy consumption is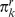 in the cloud data center. Similarly, it is of great ease to deduce the meaning of 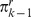. This paper can obtain these parameters of 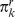 through utilizing simulation platform in the following experiments. Thus far, the incremental energy consumption is defined by the equation (3), on account of migrating **PS [m, n, r, t+t_0_** **(k)]** as for previous migration stage **PS [m, n, r, t+t_0_** **(k−1)]**.
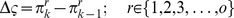
(3)The objective is optimal energy conversation and thus the following δς is supposed to be minimized to get the optimal solution obtained and it can be represented as following.
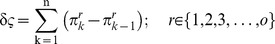
(4)Eventually, ***τ_r_*** is maximized for fulfilling performance requirements to greatest extent and then δς is minimized for better energy saving effect by the proposed PS-ES appraoch.

### 3. Performance Evaluation

An array of experiments are designed and conducted to experimentally evaluate the performance of the proposed PS-ES appraoch in this section. The energy saving effect of PS-ES from the long-term point of view and the performance on the failure rate of migration events are verified. Moreover, other two experiments are performed to make a related parameter tested and obtain the optimal power management policy, which contributes to balancing the energy cost and penalty cost due to performance violation while minimizing the total cost. The CloudSim toolkit/platform [10], as an event driven simulator, which can make it possible to calculate the total energy consumption caused by cloud data centers during the simulation period by providing a class including the methods getPower(), is utilized to simulate dynamic cloud data centers since it can create various types of entities dynamically in real time and enables to remove data center objects at runtime. These features have achieved simulating dynamic cloud environment where the varying components and entities can join, fail, or leave the system at random [10]. On the CloudSim platform, the proposed PS-ES approach is compared with random migration policy, the optimal migration policies based on PSO and the optimal migration policy PS-ABC [9] on energy consumption and the number of invalid VM migration events. Four different kinds of experiments to evaluate and test the proposed PS-ES approach have been made It can be manifested from the final experimental results that the proposed PS-ES approach has a better energy saving effect while having a better execution performance. Notably, PS-ES has presented the stability and the excellent execution performance under the circumstance of plenty of live VM migration events.

#### 3.1 Experimental Scenarios

To evaluate and test the proposed PS-ES approach, we have designed and made simulation experiments on the CloudSim platform. The simulated cloud data center is constructed as a resource pool comprising 100 hosts, which have varying computing resource available. Live VM migration requests are simulated by CloudSim. The CloudSim platform has 24 batches of VM migration requests created. Each batch accommodates 13 requests generated with different resource requirements and randomly belonging to different physical hosts. During the simulation experiments, the proposed PS-ES migration policy has been running to receive live VM migration requests while getting resource information of available physical hosts at the end of each Δt.

#### 3.2 Evaluate Energy Consumption on Varying Approaches

**Figure 1:**
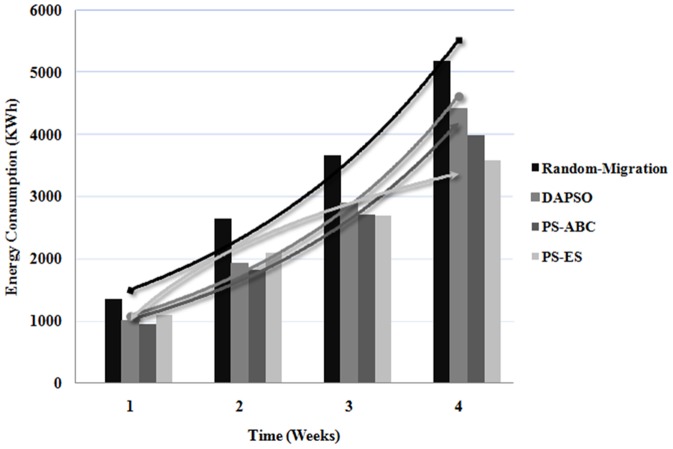
This figure has four kinds of bars, which respectively represent the energy consumption of Random-Migration, DAPSO, PS-ABC and PS-ES. The four approaches are compared in four time points on energy consumption. The trend line are also added to this figure. The amount of energy consumption is measured by KWh (KiloWatt hour).

PS-ES is compared with Random-Migration, DAPSO and PS-ABC on energy consumption in the long-term operation process of the simulated cloud data center to evaluate the efficiency and available of PS-ES in energy conservation for the placement selection of live VM migration events in the experiment scenario. In this experiment, Δt is set as 600 seconds and the migration events of each batch are uniformly distributed within an hour, which includes 6Δts. Besides, the host's resource change rate is set as 1 time per half an hour. It can be seen from **Fig. 1** that apart from the cloud data center executing Random-Migration, the cloud data center executing PS-ES consumes more energy than the cloud data centers executing PS-ABC and DAPSO after fortnight on ground that the latter two approaches have brilliant global convergence in an algorithm cycle. Although the proposed PS-ES approach isn't worse than them, it has made the migrant VMs be migrated into the corresponding optimal target hosts with a certain probability and thus to cause the result that PS-ES has a more energy consumption at the beginning stage.

As illustrated in **Fig. 1**, the cloud data center executing PS-ES has better energy conservation effect since it consumes less total energy than the other three approaches after four weeks. According to the overall trend, the fact that the cloud data center executing Random-Migration migration policy has increasingly growing incremental energy consumption between two weeks can be seen. The reason for this fact is that the Random-Migration policy doesn't enable to self-adapt to the dynamic cloud environment and thus it is likely to result in that more migration events are generated while picking out inappropriate target physical hosts in energy consumption at random.Since the cloud data centers executing DAPSO and PS-ABC have the migrant VMs migrated to the optimal target hosts for energy conservation in an algorithm cycle, they have the relatively less total energy consumed in the first fortnight. They, however, have only made the current problem optimized rather than taken this proposed problem into consideration from the perspective of cloud data centers' long-term operations. Accordingly, their current optimization is very likely to make the next problem cause more consumption and harder to optimize. On the other hand, the proposed PS-ES appraoch hasconsidered the proposed energy-saving problem from the higher perspective rather than solely been limited to achieving the global optimization of the current problem cycle by heuristic algorithms such PA-ABC.

#### 3.3 Test the Failure Rate in VM Migration Events

**Figure 2:**
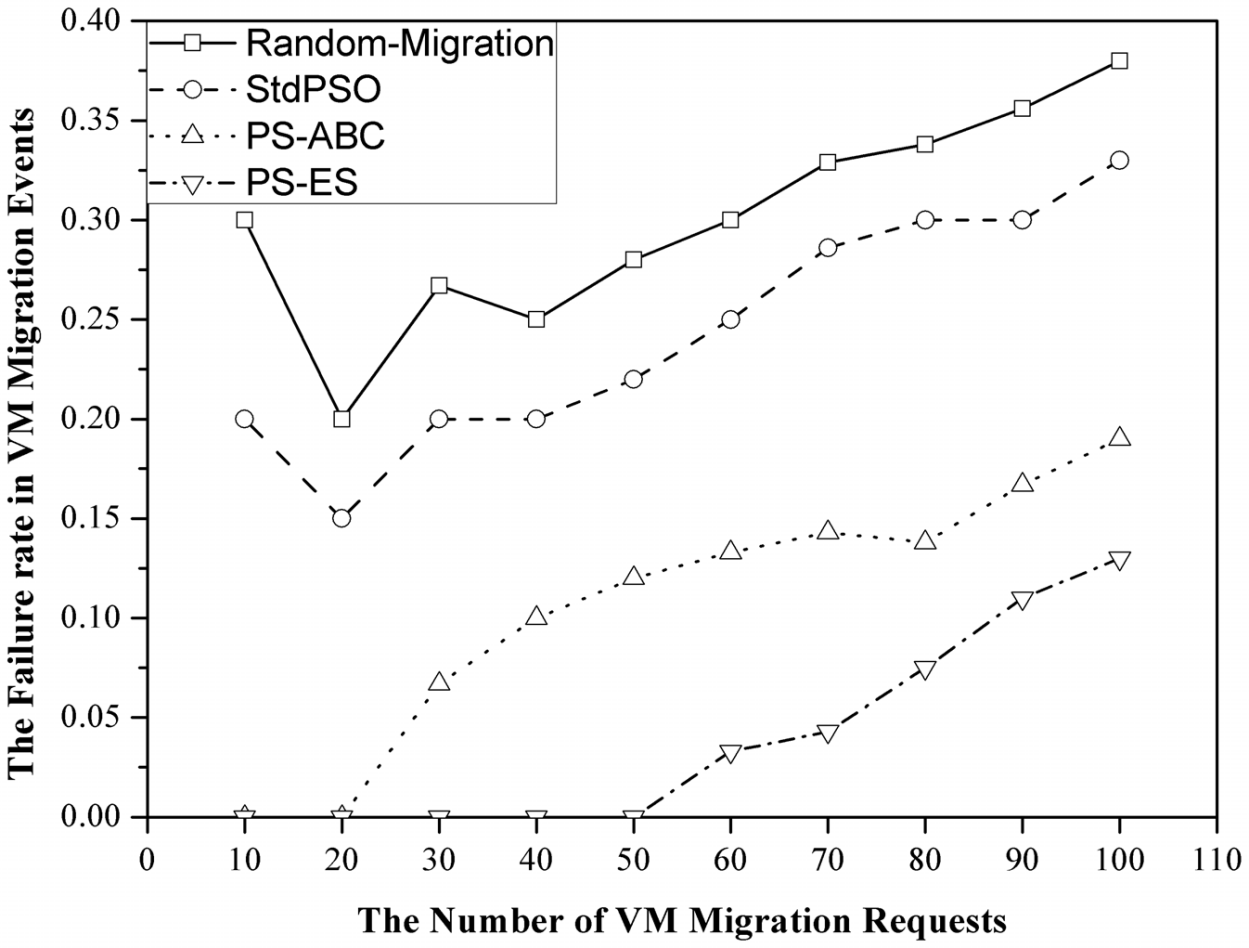
This figure consists of four kinds of curves, which respectively represent the failure rate of VM migration events of Random-Migration, StdPSO, PS-ABC and PS-ES. X axis denotes the number of VM migration requests. Y axia denotes the failure rate in VM migration events. The failure rate is equal to that the failure number of VM migration events is divided by the number of VM migration requests.

The dynamic cloud environment is carried out by the CloudSim platform through dynamically triggering a range of shut-down events or failure events of physical hosts within the interval time of the placement selection in the experiment scenario. Quite a few failures events in VM migration may be produced on account of the corresponding physical hosts' non-availability. The proposed PS-ES approach is compared with Random-Migration, the optimal migration policy achieved by StdPSO and PS-ABC on failure rate in VM migration events as illustrated in **Fig. 2**. It can be seen that, compared to PS-ABC and PS-ES, greater failure rate of VM migration events is caused by the cloud data centers executing Random-Migration and StdPSO under the varying number of VM migration requests. This is because that Random-Migration and StdPSO can't have the corresponding adjustment with the environment changed and thus to result in that their memory data is outdated. Conversely, the Boltzmann selection policy and Bayes theorem are introduced the PS-ABC migration policy and consequently it has abilities to efficiently respond to changing circumstances within the interval time. However, it isn't as excellent as the proposed PS-ES approach. The fundamental reason for that PS-ABC can have relatively less failure number of VM migration events is that it has the excellent optimization and iteration mechanisms and isn't that it aims at achieve this goal. By contrast, it is to self-adapt the dynamic cloud environment that the proposed PS-ES policy utlizes the DAPSO idea. Beside, the introduction of the SA method with the probability idea in PS-ES makes it have higher adaptability. Obviously, the proposed PS-ES should be much more efficient than PS-ABC in detecting the host failures during the interval as well as have a fit adjustment in a better manner by dynamically searching out the new available hosts that can meet the resource requirements of the VM migration requests.

#### 3.4 Comparison of the Failure Rate in VM Migration with Fixed and Variable Evaporation Factor

**Figure 3:**
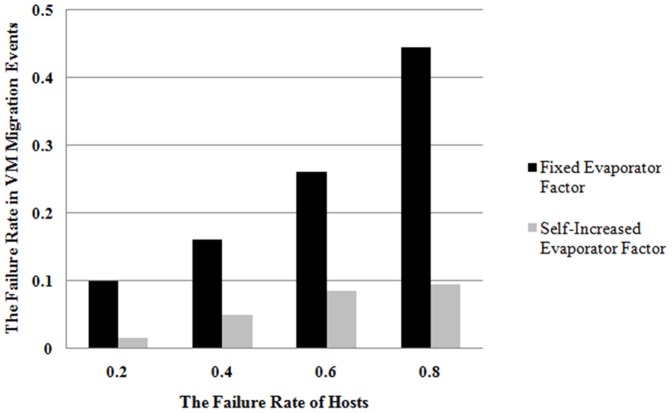
This figure has two kinds of bars, which respectively represent the failure rate on VM migration events in PS-ES with fixed evaporator factor and self-increased evaporator factor.

In this experimental scenario, we will evaluate one of the most important parameters of PS-ES, the evaporation factor **Z** which makes the proposed approach self-adaptive placement selecting in a dynamic cloud environment. As shown in **Fig. 3**, the experiment shows the impact of fixed and self-increased evaporation factor on the performance of PS-ES in a highly dynamic cloud resource pool. With the rising of the rate of failure of hosts, the rate of failure in VM migrations is recorded. The runs will be tested with fixed evaporation and self-increased evaporation factor. For the fixed evaporator factor, since the capability with which PS-ES adapts to a dynamic environment is also within a certain range, the number of failure in VM migration events will increase with the rising of the rate of failure of hosts. Whereas for the self-increased evaporator factor, the capability with which PS-ES adapts to a dynamic environment is getting stronger and thus can be aware of the failure of hosts and make the rate of failure in VM migrations lower. It shows that the adjustments in evaporation factor according to the rate of resource changing in a dynamic cloud environment reduce the rate of failure in VM migrations.

#### 3.5 Test the Incremental Energy Consumption under Different Load

**Figure 4:**
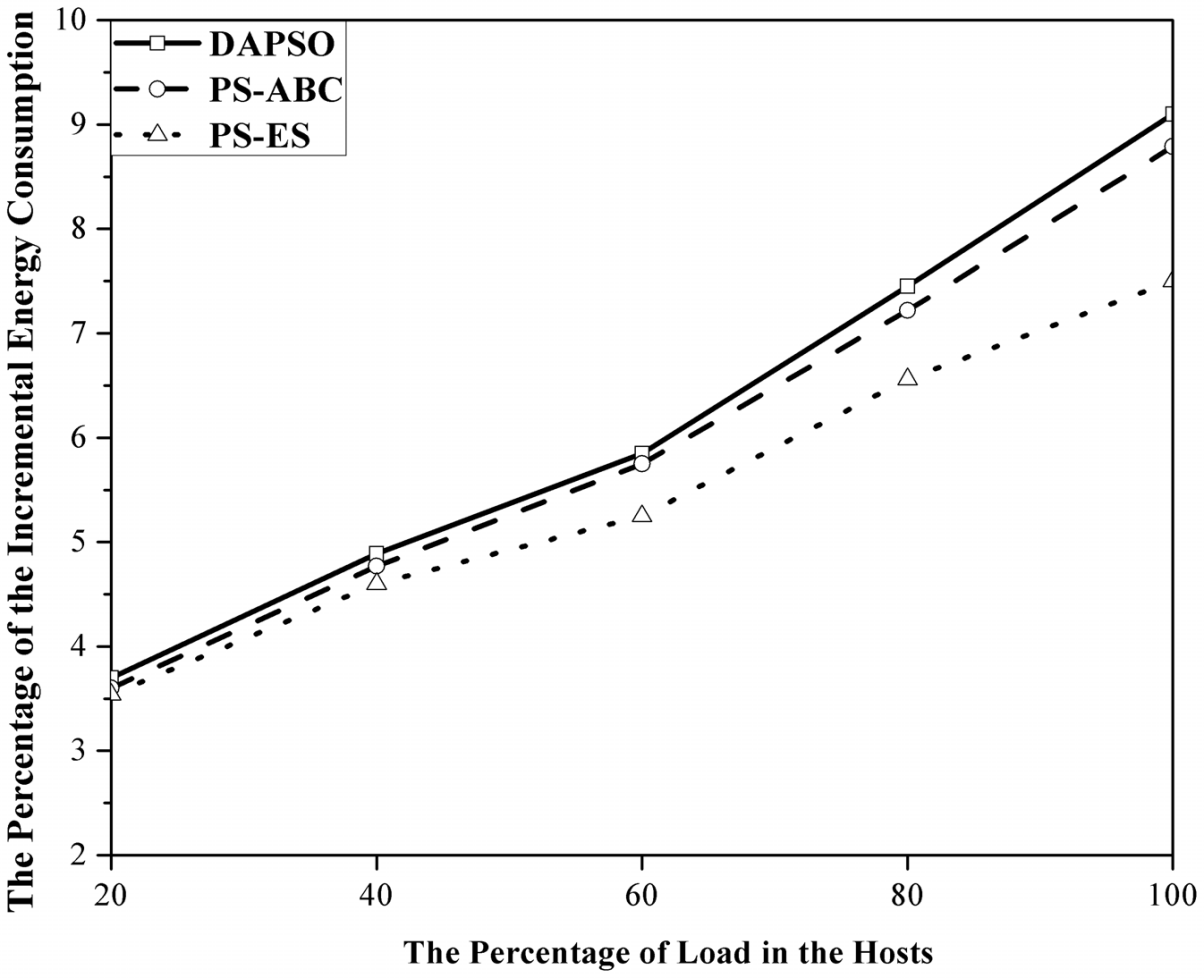
This figure has three kinds of curves, which respectively represent the percentage of the incremental energy consumption of DAPSO, PS-ABC and PS-ES with the percentage of load in the hosts increasing. X axis denotes the percentage of load in the hosts. Y axis denotes the percentage of the incremental energy consumption.

The incremental energy consumption of DAPSO, PS-ABC and PS-ES is compared in a cloud data center with varying percentage of load in this experimental scenario. The load mentioned in this experiment does not refer to the load in a physical host but the load of the whole cloud data center. In **Fig. 4**, X axis denotes the varying percentage of load in the same cloud data center while Y axis represents the percentage of the incremental energy consumption. It can be observed in **Fig. 4** that PS-ES has the relatively less incremental energy consumption than that of PS-ABC and DAPSO under increasing load. PS-ABC and DAPSO have nearly identical performance in the experiment since their efficiency and optimization degree of processing large-scale problems are similar in the cloud data center. It is well-known that the heavier the load is, the greater impact the solution of the current problem has on the future problem. Obviously, PS-ES not only has better optimization effect in the current problem but also makes better preparation for the coming problems than PS-ABC with no such excellent features in it since it has achieved the SA idea.

#### 3.6 Evaluate Power Management Policies

**Figure 5:**
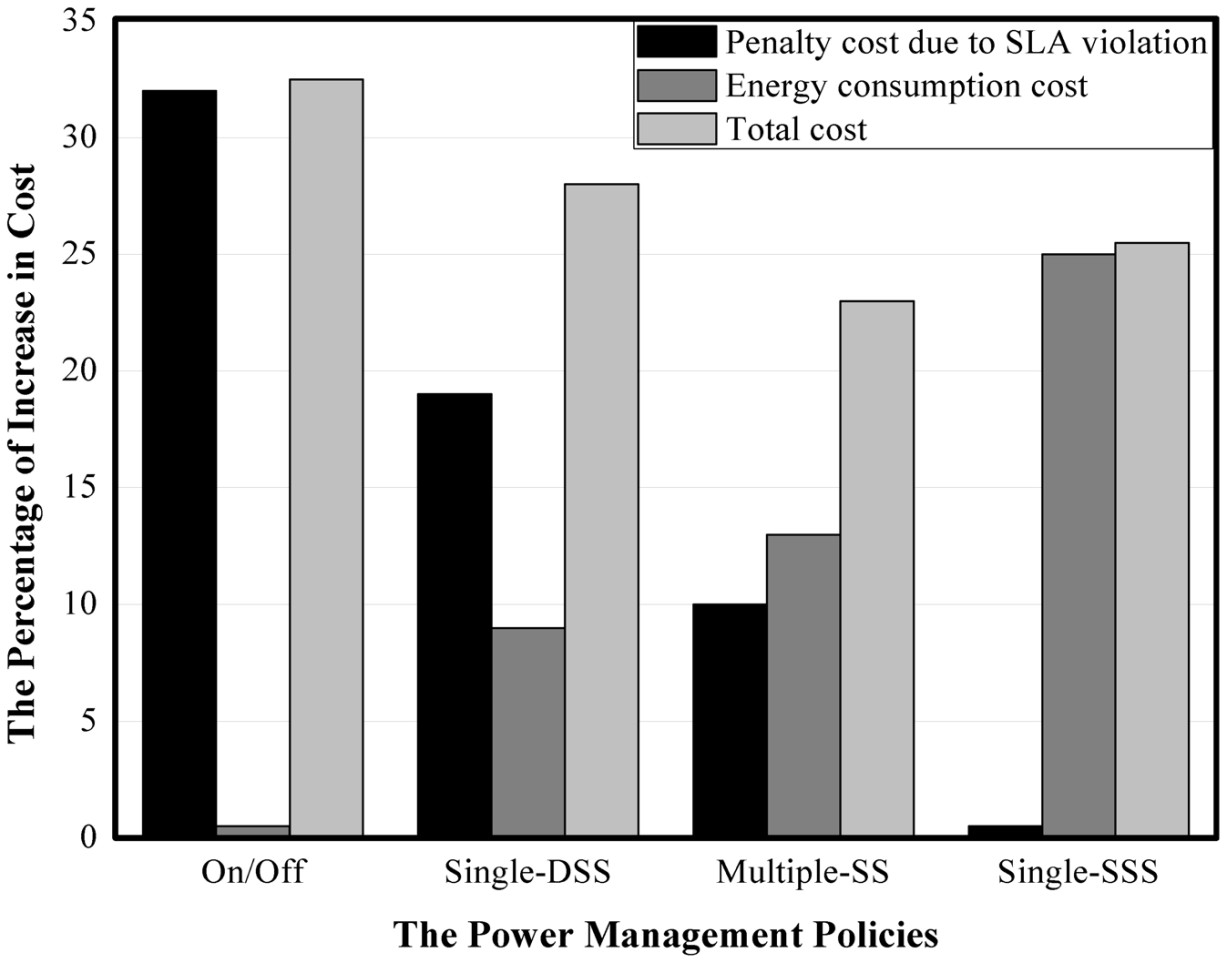
This figure has three kinds of bars, which respectively represent the percentage of increase on penalty cost due to SLA violation, energy consumption cost and total cost under varying power management policies of On/Off, Single-DSS, Multiple-SS and Single-SSS.

The auxiliary experiment is presented and conducted to pick out the optimal power management policy which has a tradeoff between the energy cost and penalty cost due to SLA (Service Level Agreements) while having abilities to relatively minimize the total cost of energy cost and penalty cost of performance violation in the cloud data center implementing PS-ES. Since live VM migration requests are time critical VM events and the cloud service provider should meet strict SLA compliance, any violations in SLA will cause penalty cost based on performance loss of VMs on the provider of a cloud data center. It can be well-understood that the performance loss is likely to be caused by energy conservation, live VM migration events, lack of network bandwidth and throughput etc. This paper, however, has not focused on these problems directly.This experiment is performed to solely find out a better power mangement policy applicable to the cloud environment implementing the proposed PS-ES to balance the cost of energy consumption and penalty cost due to SLA violation and thus to make the total cost minimized. There are, generally, four power policies available for cloud data centers to select from. As is well known, they are On/Off, Single-DSS, Multiple-SS and Single-SSS respectively. As illustrated in **Fig. 5**, the Multiple-SS power management policy leads to the better tradeoff between energy cost and penalty cost as well as have the relatively better total cost of both them. The experimental result can be analyzed. For the On/Off policy, all idle hosts are switched off. Obviouly, it can give the optimal energy saving effect but causes the high penalty cost. The single-DSS policyswitchs all idle hosts to deep sleep state, where an increase in the energy consumption cost is caused. Although the penalty cost declines significantly, its tradeoff effect is insufficient and thus the total cost is relatively more. Single-SSS makes all idle hosts switched to shallow sleep state. Almost no penalty cost is generated as SLA violation is absent in this policy. On the contrary, the policy causes the enormous increase in the energy consumption cost. The Multiple-SS policy keeps some of the idle hosts in deep sleep and others in shadow sleep state according to a short-term prediction technology. It can dynamically switch the state of a physical host in terms of the workloads in real time in cloud data centers. To sum up, the Multiple-SS policy is picked out as the optimal power management policy in cloud data centers implementing PS-ES.

## Discussion

It is noticeable that there exists an interesting problem in the proposed PS-ES approach. It is obvious that a candidate solution vector of the proposed problem is supposed to be denoted as a sequence of integers that represent these candidate hosts in the cloud data center. The PSO-based approaches, however, initialize their swarms at random in PS-ES. Furthermore, the velocity function of the particles has some random coefficients which are limited between 0 and 1. Accordingly, even though each element of a particle is limited to the Integer type during the implementation, the problem that the solution vectors aren't integers still exists. To address this problem, PS-ES employs the SPV (Smallest Position Value) rules presented by in [11], [12] rather than limit all the elements to the Integer type. In a word, each particle's vector given by the classical PSO algorithm can be converted to a valid solution vector fit for the proposed problem through the SPV rules. The process of applying the SPV rules into the proposed PS-ES approach can be understood as following. First, these candidate hosts in the cloud data center are numbered from 0 to m−1. Second, after the velocity and position vectors of each particle are initialized randomly, all the elements of each position vector are sorted in ascending order and then are numbered from 0 to n−1 as well as have a modulo operation of m. A reasonable case in point may be given. Within a time window **Δt**, there are six VMs to be migrated in a cloud resource pool, in which four physical hosts are available currently. If a candidate solution vector (−1.21, 3.29, 1.26, −0.12, 4.76, 0.78) is generated in the process of iterations, it will be converted to (0, 4, 3, 1, 5, 2) firstly and then to (0, 0, 3, 1, 1, 2). At this point, the solution vector is meaningful and available. In PS-ES, all the position vectors refer to the vectors which the original vectors have been converted to according to the SPV rules.

**Figure 6:**
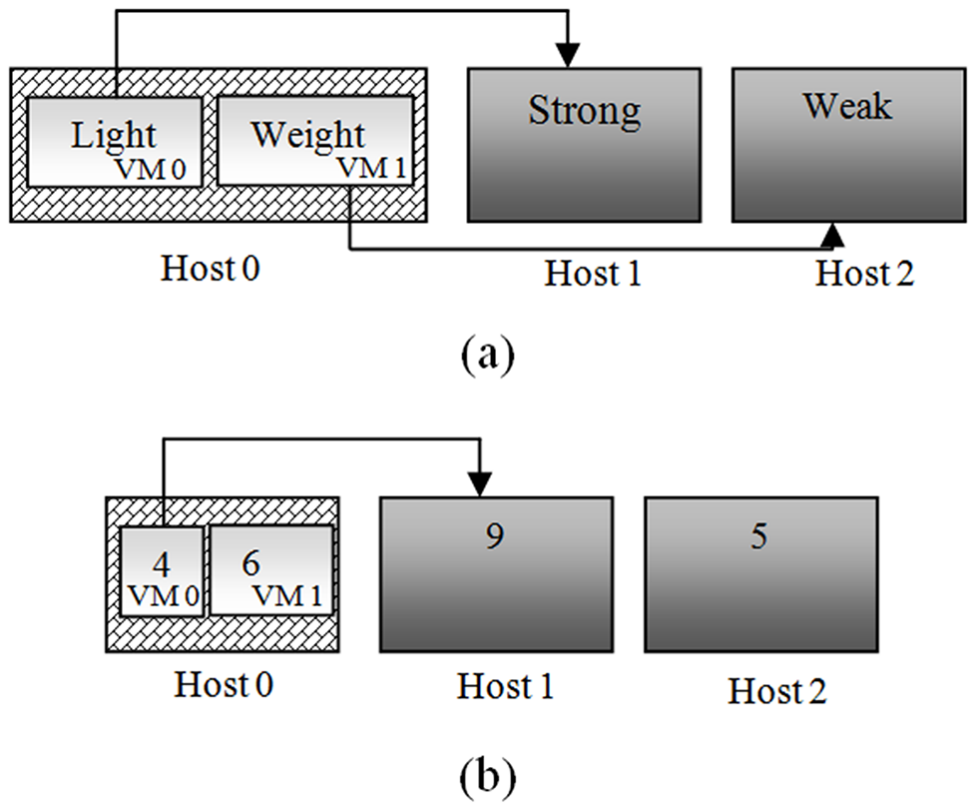
This example has shown two kinds of live VM migration situations. In (a), the situation represents that in cloud data centers there exists the fact that sometimes the VMs with the weight are migrated to the hosts with weak computing power in contrast to the VMs with the light, which are migrated to the hosts with strong computing power. In (b), the situation is similar to that in (a).

Starting from this intuition, the PS-ES algorithm which aims at live VM migration events gives consideration to both a short-term optimization and a long-term optimization to save more energy. To achieve this goal, the proposed approach utilizes the improved PSO algorithm to search this global optimal solution in order to optimize the current problem situation. And then it takes use of SA idea to optimize the future problem situation. To illustrate our problem, we give an example as shown in **Fig. 6**.

In (a), a resource pool consists of three hosts. The rated power of all the hosts is the same. There are two VMs running in host 0. In the first time window **Δt**, VM 0 with a light computing workload in the host 0 sends a migration request for some reason. The current problem caused during the first **Δt** is finding out the optimal target host of VM 0 to minimize the increment energy consumption. As is well known, the host node with a larger ‘performance per watt’ will consume less energy in the case of dealing with the same load. While the percentage of load of a host is beyond a critical value, it will reach its performance bottleneck. After that, the host's performance will degrade significantly during dealing with the next load. The consumed energy will increase in the case of dealing with the same load. Therefore, the host with a stronger computing power which shows that it has a better ‘performance per watt’ in the case that the rated power is the same will cause the less increase of energy consumption. Therefore, the optimal solution of the current problem is host 1. That is, VM 0 will be migrated to host 1 optimally. After the second time window **Δt**, VM 1 needs to be migrated. At this time, if the host 1 has not been able to accommodate it, it will have to be migrated to host 2 with the weak computing power. We know that the energy consumption is to increase rapidly when a host with weak computing power deals with a relatively heavy workload. In other words, starting from the time when a host begins to feel the pressure caused by dealing with its workload, the energy consumption caused by the host will suddenly increase rapidly and be significantly affected by the increase of workload. In fact, if VM 0 is not migrated onto host 1 but onto host 2 after the first time window **Δt**, VM 1 will be migrated onto host 1. At this moment, both host 1 and host 2 won't feel the pressure during dealing with their own workload. It is obvious that the latter incremental energy consumption is less from a long-term point of view.

In (b), we give a specific instance in order to further illustrate another situation of this problem. The numbers in VM 0 and VM 1 represent their required computing resource and the numbers in host 1 and host 2 represent their available computing resource. After a time window **Δt**, VM 0 needs to be migrated for some reason. The current problem caused during the first **Δt** is finding out the optimal target host of VM 0 to minimize the increment energy consumption. Host 1 has stronger computing power as it has more computing resource. As previously mentioned, the optimal solution of the current problem should be host 1. That is, VM 0 will be migrated to host 1 optimally. After the second time window **Δt**, VM 1 needs to be migrated. At this point, the available resource of host 1 and host 2 isn't enough to accommodate VM 1. This situation will cause a new migration event that VM 0 is migrated from host 1 to host 2. This will increase energy consumption and cost. The incremental energy consumption caused by a new migration event is much larger than that caused by only running a VM. This is because a VM is always running regardless of migrating or not. The migration cost has caused a net increase of energy consumption. Thus, if VM 0 is migrated onto host 2 after the first **Δt**, the result will be better after the second time window **Δt**. In another word, the host 1 is a global optimal solution for the current situation. However, it is a local optimal solution for a long-term view. After all, our goal is to achieve better energy saving during the long-term operation of a cloud data center but not only the current and short-term energy saving. After realizing these facts, under the background that the future is unpredictable we think of taking advantage of the SA idea to further optimize this problem. Let the VMs be probabilistically moved to suboptimal host which may be its optimal host from a long-term view in the future. From the long-term and global point of view, compared to optimally migrating and randomly migrating, this approach can further minimize the increment energy consumption.

To achieve this idea of the proposed PS-ES, the global search of optimal solution is still important at the end of every time window **Δt** as it is the research basis of the problem. It firstly should have ability in finding the optimal solution for the current **Δt**. And then it can proceed further. We employ the PSO algorithm to search the optimal solution. Thus, one can see that the PSO-based algorithm is our main algorithm. To improve the performance and accuracy of the PSO algorithm, we also introduce the SA idea into the PSO algorithm to optimize its global search and avoid being trapped into the local optima. Generally, the PSO algorithm only returns a global optimal solution at last. However, in our proposed approach we need not only the global optimal solution but also the available local optimal solutions. Moreover, we also need obtain the reasonable probability value of each solution. To address the two problems, we take two reasonable, effective and simple ways. Based on the PSO's characteristics that the global best vector of every round of iteration actually represents a solution of every round of iteration and is gradually converged to the optimal solution of the last round of iteration, the last several rounds of global best solution vectors can be regarded as the optimal solution and several suboptimal solutions. Thus, we present a parameter **N**. The PS-ES will preserve the last **N** global best vectors of the PSO-based algorithm to obtain the optimal solution and the available suboptimal solutions. Put simply, the PSO process of the proposed approach doesn't return a solution vector but a solution matrix. At this time, each migrant VM has obtained its **N** solutions which constitute a column vector of that matrix. For any VM, the majority of these solutions should be the optimal solution and the minority should be the suboptimal solutions as well as few solutions may be the unreasonable solutions. Subsequently, for a VM, the probability of its each solution is obtained by calculating its frequency according to the Probability Theory and Mathematical Statistics. To filter out the individual unreasonable solutions, the Expectation and Standard Deviation are calculated. If the Absolute Value of the difference between a solution and the Expectation is larger than the Standard Deviation, the solution should be filtered out. The sum of the probabilities of all the unreasonable solutions is assigned to others solutions proportionally. Now, the optimal solution, the suboptimal solutions and their probabilities have been obtained. To achieve a probabilistic migration, we utilize a probability wheel and a random pointer. The greater probability will have a larger sector which the pointer is more likely to stop within. In a specific implementation, each sector is represented as an interval **(a, b]** included in **[0, 1]**. The pointer is represented as a random number between 0 and 1. The solution which the interval which the random number belongs to represents is the final solution of a VM. Each VM has found its target host of live migration so far. Each target host is found neither randomly nor for the current optimization but for achieving a long-term optimization on energy saving according to the SA idea. Thus, compared to them, the PS-ES approach is more efficient and meaningful.

In a cloud data center, there are many functional modules and system architectures running together for the different purposes. Many systems are managing the cloud environment at the same time. So, for each system, the environment and resource which they are facing are dynamic. During the running of an algorithm of any system, its objects and environment may have been changed. Therefore, the design of the PS-ES algorithm should adapt to a cloud environment with dynamic resource. The PS-ES employs a dynamic adaptive PSO (DAPSO) for monitoring and automatically reacting to the changes in a cloud environment. DAPSO is proposed in [13] to search out the changing optimal solution in a dynamic and noisy cloud computing environment. It doesn't need any centralized control and only needs to update the memory of each particle. DAPSO has achieved dynamically adapting to the changed cloud environment by adjusting personal best fitness value of each particle. In the classical PSO, each particle will compare the fitness value of its current location with that of its personal best location in each round of iteration. If the current fitness value doesn't have any improvement, its personal best fitness value will not be changed; if not, the personal best fitness value will be changed to the current fitness value. Nevertheless in the DAPSO, if the current fitness value doesn't have any improvement, its personal best fitness also will be changed according to the equation (5).
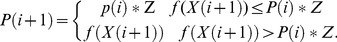
(5)The parameter **Z** is called the evaporation factor and its value is between 0 and 1 [14]. Each particle has the same evaporation factor. However, the updating frequency of each particle may be different. The past personal best fitness value and the current fitness value of a particle determine its updating frequency. Its main idea is that if the current fitness value of a particle continuously doesn't have any improvement through using its previous searching experience, its personal best value will gradually be evaporated and decrease at the rate of the evaporation factor **Z**. Eventually, the personal best fitness value will be lower than the fitness value of the particle's current location and the best fitness value will be replaced by the particle's current fitness value corresponding to the changed cloud environment [15]. The fitness value which in the changed environment is generated later will become its personal best fitness value by using the evaporation factor. For doing so, the PSO algorithm can be self-adaptive for the changing of cloud environment and makes the particles respond the changing of cloud environment as well as make the PSO process match the reality. Besides, it has ability in sensing the new powered on hosts and recently powered off hosts and considering this updated information. Thus, it can also optimize our proposed approach further.

In the proposed PS-ES, there are many parameters, most of which have great influences on the proposed algorithm. The number **MaximumofIteration** of iteration of the PSO process and the number **N** of the returned solution vectors are two important parameters and their values are related to the efficiency of PS-ES. For instance, if **MaximumofIteration** is too large and **N** is too small relatively, the probability with which the optimal solution is the final solution is closed to 100%. It will make the SA idea invalid in our approach. On the contrary, if **MaximumofIteration** is not large enough and **N** isn't small relatively, several suboptimal solutions may appear and each has a big probability. This will make our approach inefficient and inaccurate as well as make the proposed idea invalid. Therefore, the two values need to be researched further to optimize the algorithm. In the proposed approach, **MaximumofIteration** is set to 200 and **N** is set to 50. Also, the initial temperature **T** of the SA and its attenuation factor **α** are another two important parameters. **T** should be initialized to an enough big value and it makes the corresponding algorithm have a stronger global search in the early iteration. Then **T** is gradually decreased by using **α**, as makes the search process gradually converged to an optimal solution in the later iteration. Thus, the values of **T** and **α** are important for the SA idea. They should be set to the appropriate values as much as possible.

Let us consider the obtained probability values of solutions mentioned above in the view of Probability Theory and Statistics. By many direct observations and experiments, we have discovered that for any VM, the Probability Distribution of its solutions yields to random Poisson distribution. Here we will analyze this phenomenon theoretically. Firstly we know that when a random event occurs at a fixed average instantaneous rate **λ** randomly and independently, the number of occurrences of this event within a unit time should approximately yield to Poisson distribution. From a long-term view, the migration event of some VM occurs at a relatively fixed average instantaneous rate randomly. For this VM, each available target host is independent each other and the probability with which any available host is selected as the target host should be the same. Therefore, if a VM is migrated according to the traditional policy of random migration, from the view of a long-term operation (A large number of **Δt**) of a cloud data center, the number of times for which a VM has been migrated to a certain host should approximately yield to Poisson distribution. Similarly, for the PS-ES approach, in a time window **Δt**, each VM has a solution space whose size is **N**. It can be easy to deduce that in the solution space, the number of each solution should also yield to Poisson distribution. Thus, the obtained probabilities values above approximately yield to Poisson distribution in the probability figure. From the side, this fact also shows the mathematical background and theoretical basis of the proposed problem and PS-ES approach as well as further demonstrates its rationality.

## Methods

### 1. The Proposed System Architecture

**Figure 7:**
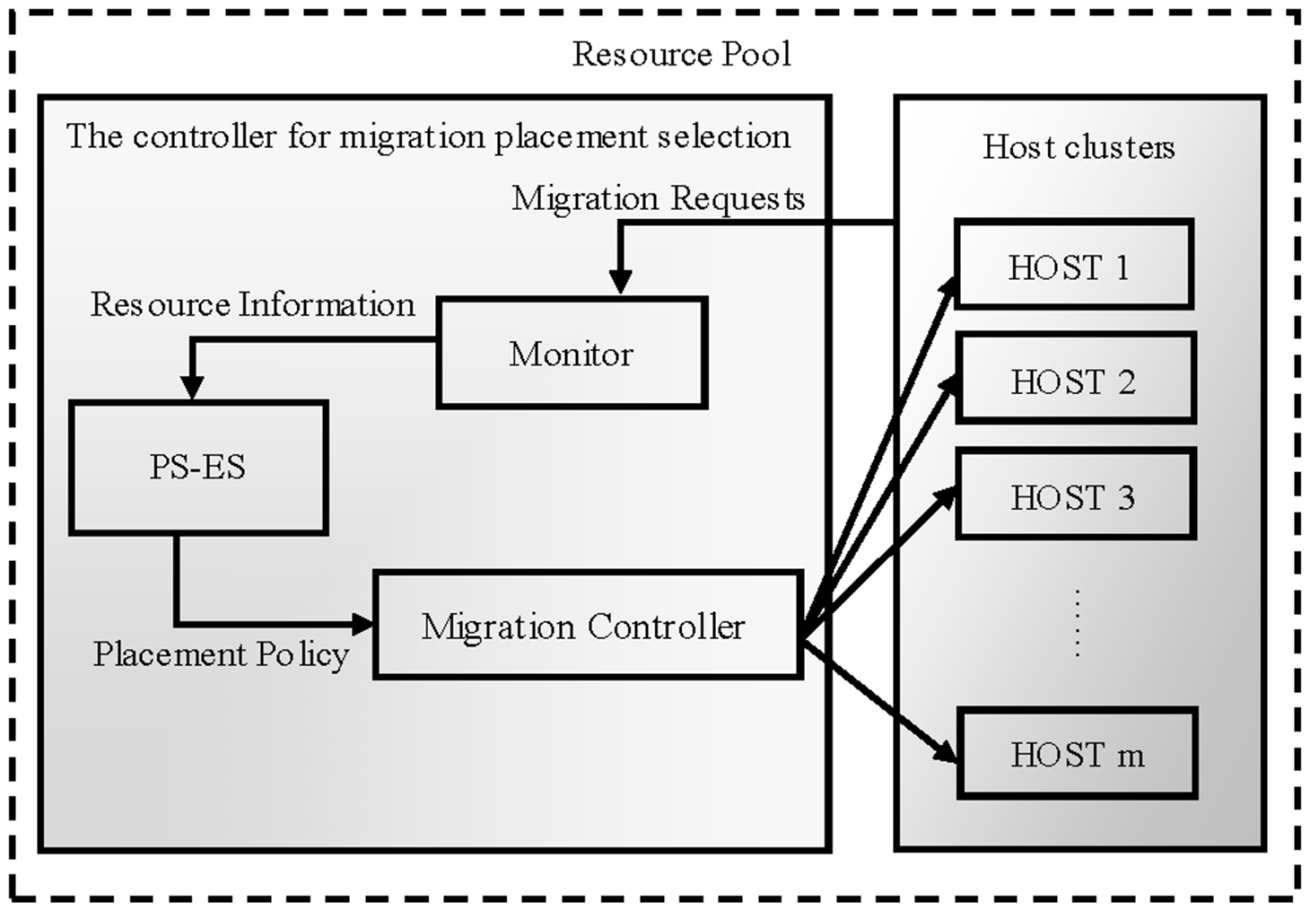
In this figure of PS-ES's architecture, the right part represents the set of available physical hosts, where a plenty number of VMs are running. In the left part, the controller consists of three modules of Monitor, PS-ES and Migration Controller. It implies that the proposed architecture's processing flow.

In the paper, the system architecture of PS-ES is proposed as illustrated in **Fig. 7**, in which the position of the controller PS-ES for migration placement selections can be seen and its interaction with other entities is clear [16]. Within a time window Δt, live VM migration requests will be accumulated by the Monitor while the current available amount of computing resource such as CPUs, memory, storage and network bandwidth etc. as well as energy consumption is updated. After a time window Δt, these information will be transferred to the PS-ES controller, where the placement policy is supposed to be generated by utilizing the proposed PS-ES approach and obtained information. Subsequently, the generated migration solution will be sent to Migration Controller, which is in charge of executing live migration of these VMs. Eventually, the VMs are migrated into their target hosts.

### 2. Solution Representation

Aiming to achieve an efficient PSO-based approach for picking out the optimal vector of target hosts of all migrant VMs accumulated within a time window Δt, the problem of solution representation is the primary problem since it reflects a direct relationship between the proposed problem domain and the proposed approach [11]. It is well-known that when applying a PSO-based approach to solve a problem, a particle is generally denoted as a solution of the specific problem. In this paper, the proposed problem is concerned about migrating **n** VMs into **m** physical hosts and is an **n**-dimensional problem. Thus, a particle (solution) can be represented as an **n**-dimensional vector. Each of its elements has a discrete set of possible placement selection and is limited to **m**. In the proposed PSO-based approach, the position vector is denoted as 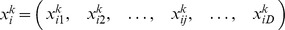, where 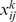 represents the position of ***j***th dimension (the ***j***th migrant VM) of ***i***th particle (the ***i***th possible solution vector) in ***k***th generation. The velocity vector is denoted by 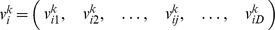, where 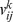 represents the velocity in ***j***th dimension of ***i***th particle in ***k***th generation. The personal best position vector is denoted by 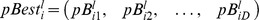 and represents the ever best position of each particle so far. The global best position vector is denoted by 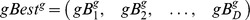 and represents the obtained best position of the entire population so far.

### 3. The Main Idea of PS-ES

Its main idea can be divided into two aspects. On the one hand, from the point of view of a time window **Δt**, we introduce the SA idea into the PSO algorithm to find out the better global optimal solution and improve the accuracy of PSO global search. On the other hand, from the point of view of a long-term operation of a cloud data center, we have found the fact that the optimal placement searched out in the current time window may be a local optima in a long-term process as the next some VM may be more suitable this host. However, we can't predict the future VM migration requests. Thus, we again introduce the SA idea into the long-term process. That is, the migrant VM is not necessarily moved to the optimal host but may be moved into some suboptimal host with a certain probability for achieving a long-term optimization. To achieve this optimization, the probability theory and the probability wheel idea is also introduced to the proposed approach.

### 4. The Implementation of PS-ES

In this section, we describe the specific process of PS-ES. Details are as follows:

#### Find the optimal target host of each VM

the PS-ES module receives all required information and begins running the algorithm. First, the parameters required by the approach are initialized. The PSO performs the first round of iteration. The initial velocity vector and position vector is initialized randomly. The fitness value of each particle is obtained by using the fitness function (1) & (2). The PSO sets personal best position vector 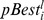 of each particle as its current position and sets personal best value of each particle as its current fitness value. The PSO sets global best position vector 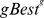 of the population as the position where the particle has corresponding best personal best value among all the particles and sets global best value as the corresponding fitness value. Second, the PSO begins performing the loop of iteration process from the second round of iteration. Each particle updates its velocity and position by using the formulas of PSO but isn't moved to the new position temporarily. The variation **ΔE** of the fitness value between the next position and the current position is obtained for the SA. If the variation **ΔE** is larger than or equal to 0, the particle should be moved to the new position; if not, according to the SA idea, the particle neither is moved to the new particle undoubtedly nor isn't moved to the new position undoubtedly but is moved to the new position with the probability of **exp (ΔE/KT)**. Subsequently, each particle is annealed and cooled. That is, **T** is attenuated as **αT**, where **α** is the attenuation factor. Now the position of each particle has been fixed in the current iteration, so each particle compares its current fitness value with its personal best value. If better, updates it; if not, does nothing. If there is more than one particle with maximum fitness value, the final particle with the minimum value of function (4) is found. Subsequently, the global best value is updated. The inertia weight ***ω*** is updated as ***ω*** *exp(−iteration). If the termination condition of the maximum number **MaximumofIteration** of iteration is met, the loop of iteration ends; if not, the algorithm begins the next round of iteration. Finally, when it ends, the particle with global best value is the solution particle. In another word, the global best vector 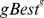 is the solution vector.

#### Find the final placement of each VM

**Figure 8:**
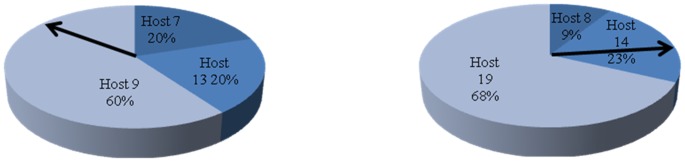
The figure is presented to show how to utilize the SA idea to PS-ES. These two pie charts describe that PS-ES obtains the final target host of a VM by selecting the solutions with a certain probability value.

in order to obtain some available suboptimal solutions, the approach will record the last **Ν** global best vector of the last **N** rounds of iteration of the PSO algorithm. We can regard the **N** vectors as an N*n of matrix, each of the **n** column vectors of which represents **N** solutions of the place selection of the corresponding VM. We consider that the **N** solutions consists of the optimal, the suboptimal and unreasonable solutions. The unreasonable solutions ought to be very few and may also not exist. For a migrant VM, we can find a probability of every kind of solution by using the **N** solutions. And the expectation and standard deviation of the solutions are also found by using the equations (6) & (7).
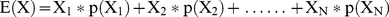
(6)

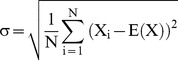
(7)One can find out the reasonable solution by using the expectation and standard deviation as well as assign the probabilities of the unreasonable solutions to other solutions proportionately. If the sum of the probabilities of all unreasonable solutions is ***M***, the probability of each available solution is converted to **1/1−** ***M*** times as the original probability. At this point, each migrant VM has a probability wheel as shown in **Fig. 8**. A pointer randomly rotates on the probability wheel. The host which the area where the pointer finally stops represents is the final target host. In the specific implementation, a random number limited between 0 and 1 can be used to find the final target host by finding the range within which the random number is. It is understandable that the probability of the optimal host is much larger than that of the suboptimal hosts. However, each migrant VM may be moved to suboptimal host with a certain probability. It is obvious that with the SA idea the approach is more efficient than randomly migrating and optimally migrating from a long-term's point of view as it can be understood as reaching a compromise between migrating optimally and randomly as well as achieving a long-term optimization relatively. At last, each migrant VM has the final target host. An **n** dimension of solution vector is returned.

## Conclusions

In this paper, a novel placement selection policy of live VM migration PS-ES is proposed and we give its main idea, implementation and evaluation. It employs the improved PSO-based approach and the Simulated Annealing idea. In the improved PSO-based approach, we introduce the limitation of maximum velocity, the adaptive adjustment policy of inertia weight **ω**, the dynamic adaptive PSO idea and simulated annealing idea into the classic PSO algorithm to achieve optimizing the PSO algorithm to search out the global optimal solution. In the proposed PS-ES, we have utilized the Simulated Annealing idea for twice. For the first time, it is used to optimize the PSO process to improve the performance and efficiency. For the second time, it is as the second part of the PS-ES approach for optimizing the whole approach. Also, aiming to connect the improved PSO-based algorithm and simulated annealing process, we take use of the Probability Theory and Mathematical Statistics as well as the characteristics of the algorithm itself to obtain and process data. We have discovered a noteworthy theoretical phenomenon that the probability values which are used for the SA idea approximately yield to Possion distribution. It has shown that the proposed PS-ES approach has the strong mathematical basis. PS-ES achieves the high-efficiency of energy consumption and the stability of requirement performance. It not only minimizes the incremental energy consumption of a cloud data center but also minimizes the number of failure in VM migration events relatively. What is more, it aims at achieving the better energy conservation from a long-term view of operation of a cloud data center and protects the performance of VM running. In the proposed PS-ES approach, there are some open problems which need be studied further and experience problems which need many experiments to gradually get a better solution. The **T** value of the SA idea is an experience problem and needs perform several experiments to obtain a fit value with the condition that **T** makes **exp(−ΔE/KT)** close to 1 at the beginning. The **N** value of our approach is also an experience problem. Its situation has been clarified above. It is set to 50 in this paper. Both the attenuation factor **α** value and the evaporation factor **Z** value are the open problems.

To evaluate the PS-ES approach, we have conducted several experiments on the CloudSim platform. Firstly, in the comparison experiment of Random-Migration, DAPSO, PS-ABC and PS-ES in Energy Consumption, the PS-ES approach has the least incremental energy consumption from a long-term view of operation of the cloud data center. Secondly, in the comparison experiment of the number of failures in VM migration events, the PS-ES approach has the less number of failures in VM migration events than that of random migration and optimal migration. Thirdly, in the comparison experiment of the failure rate in VM migration with fixed and variable evaporation factor, the result has shown that the increased evaporation factor has the better capability to adapt to a dynamic cloud environment. Eventually, in the experiment of testing the incremental energy consumption under different load, the result has shown that under both the light and heavy load, the PS-ES approach shows the relatively better energy saving effect. Aiming to get the optimal policy management policy which can balance the energy cost and penalty cost due to performance violation in cloud data centers executing PS-ES, an auxiliary experiment is designed and performed and proved that the multiple-SS policy is supposed to the best selection. It can be observed through the final experimental results that PS-ES, as a placement selection policy of live VM migration for energy conservation, is efficient and have a great significance towards green cloud data centers.

In the next work, we plan to further study energy saving optimization for live VM migration policy with such optimization processes that as many physical hosts as possible are shut down. Subsequently, we will take the network situation into consideration in the process of picking out physical hosts. The connectivity between physical nodes will be studied. Furthermore, since the PS-ES appraoch proposed in this paper is aiming to the local domain, we intend to extend the PS-ES idea to several cross-domain cloud data centers so as to deeply match the demanding in real world.

## References

[1] Barham P, Dragovic B, Fraser K, Hand S, Harris T, et al (2003) Xen and the Art of Virtualization. ACM SIGOPS Operating Systems Review 37 5: 164–177.

[2] Li Y, Li W, Jiang C (2010) A Survey of Virtual Machine System: Current technology and future trends. Proceedings of the 3th International Symposium on Electronic Commerce and Security. Guangzhou, Guangdong: IEEE press. pp. 332–336.

[3] Armbrust M, Fox A, Griffith R, Joseph A, Katz R, et al (2010) Above the Clouds: A View of Cloud Computing. Communications of the ACM 53 4: 50–58.

[4] Rusu C, Ferreira A, Scordino C, Watson A (2006) Energy Efficient Real-time Heterogeneous Server Clusters. Proceedings of IEEE Real-Time and Embedded Technology and Applications Symposium. San Jose, California: IEEE press. pp. 418–428.

[5] Srikantaiah S, Kansal A, Zhao F (2009) Energy Aware Consolidation for Cloud Computing. Energy 10 20: 30–40.

[6] Verma A, Ahuja P, Neogi A (2008) PMapper: Power and Migration Cost Aware Application Placement in Virtualized Systems. Proceedings of the 9th ACM/IFIP/USENIX International Conference on Middleware. New York: Spinger-Verlag. pp. 243–264.

[7] Li B, Li J, Huai J, Wo T, Li Q (2009) EnaCloud: An Energy-Saving Application Live Placement Approach for Cloud Computing Environments. Proceedings of IEEE International Conference on Cloud Computing. Bangalore, India: IEEE press. pp. 17–24.

[8] Jeyarani R, Nagaveni N, Vasanth RR (2011) Self Adaptive Particle Swarm Optimization for Efficient Virtual Machine Provisioning in Cloud. International Journal of Intelligent Information Technology 7 2: 25–44.

[9] Gaochao X, Yan D, Jia Z, Liang H, Xiaodong F (2013) A Novel Artificial Bee Colony Approach of Live Virtual Machine Migration Policy Using Bayes Theorem. The Scientific World Journal 2013 2013: 369209. 24385877 10.1155/2013/369209 PMC3872427

[10] Calheiros RN, Ranjan R, Beloglazov A, De Rose CA, Buyya R (2011) Cloudsim: A Toolkit for Modeling and Simulation of Cloud Computing Environments and Evaluation of Resource Provisioning Algorithms. Software: Practice and Experience 41 1: 23–50.

[11] Tasgetiren MF, Sevkli M, Liang YC, Gencyilmaz G (2004) Particle Swarm Optimization Algorithm for Permutation Flowshop Sequencing Problem. Lecture Notes in Computer Science 3172: 382–389.

[12] Tasgetiren MF, Sevkli M, Liang YC, Gencyilmaz G (2004) Particle Swarm Optimization Algorithm for Single-machine Total Weighted Tardiness Problem. Proceedings of 2014 Congress on Evolutionary Computation. Portland, Oregon: IEEE press. pp. 1412–1419.

[13] Carlislie A, Dozler G (2002) Tracking Changing Extrema with Adaptive Particle Swarm Optimizer. Proceedings of the 5th Biannual World Automation Congress. Orlando, Florida: IEEE press. pp. 265–270.

[14] Shi YH, Eberhart RC (1998) Parameter Selection in Particle Swarm Optimization. Proceedings of the 7th Annual Conference on Evolutionary Programming. Berlin: Springer. pp. 591–600.

[15] Hu X, Eberhart RC (2002) Adaptive Particle Swarm Optimization: Detection and Response to Dynamic Systems. Proceedings of the World Congress on Computational Intelligence. Honolulu, HI: IEEE press. pp. 1666–1670.

[16] Jeyarani R, Vasanth RR, Nagaveni N (2009) Implementation of Efficient Light Weight Internal Scheduler for High Throughput Grid Environment. Proceedings of the National Conference on Advanced Computing in Computer Applications. Coimbatore, INDIA. pp. 283–289, 2009.

